# REST/NRSF deficiency impairs autophagy and leads to cellular senescence in neurons

**DOI:** 10.1111/acel.13471

**Published:** 2021-09-14

**Authors:** Anna Rocchi, Emanuele Carminati, Antonio De Fusco, Jagoda Aleksandra Kowalska, Thomas Floss, Fabio Benfenati

**Affiliations:** ^1^ Center for Synaptic Neuroscience and Technology Istituto Italiano di Tecnologia Genova Italy; ^2^ IRCCS Ospedale Policlinico San Martino Genova Italy; ^3^ Department of Experimental Medicine University of Genova Genova Italy; ^4^ Helmholtz Zentrum München Deutsches Forschungszentrum für Gesundheit und Umwelt (GmbH) Neuherberg Germany

**Keywords:** autophagy, mitochondria, neurons, oxidative stress, rapamycin, REST/NRSF, senescence, trehalose

## Abstract

During aging, brain performances decline. Cellular senescence is one of the aging drivers and a key feature of a variety of human age‐related disorders. The transcriptional repressor RE1‐silencing transcription factor (REST) has been associated with aging and higher risk of neurodegenerative disorders. However, how REST contributes to the senescence program and functional impairment remains largely unknown. Here, we report that REST is essential to prevent the senescence phenotype in primary mouse neurons. REST deficiency causes failure of autophagy and loss of proteostasis, increased oxidative stress, and higher rate of cell death. Re‐establishment of autophagy reverses the main hallmarks of senescence. Our data indicate that REST has a protective role in physiological aging by regulating the autophagic flux and the senescence program in neurons, with implications for neurological disorders associated with aging.

## Graphical abstract

Cellular senescence is one of the aging drivers and a key feature of a variety of human age‐related disorders. The transcriptional repressor REST deficiency leads to cellular senescence in primary neurons. Rest depletion causes DNA damage, impairment of autophagic flux, mitochondrial dysfunction, and alteration of SASP genes expression. We describe a novel transcriptional regulator of the senescence program.

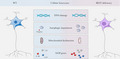

## 1 INTRODUCTION

Aging is a process characterized by the progressive accumulation of cellular changes, leading to functional deficits over time. A variety of cellular and molecular hallmarks of aging have been described: genomic damage, telomere attrition, epigenetic alteration, loss of proteostasis, deregulated nutrient sensing, mitochondrial dysfunction, stem cell exhaustion, altered cell‐to‐cell communication, and cellular senescence (Lopez‐Otin et al., 2013).

Cellular senescence is a stress response program, which is activated to arrest cell cycle and protect from uncontrolled proliferation. Acute induction of cellular senescence is considered to have beneficial effects in embryogenesis, wound healing, and tumor inhibition, whereas sustained activation of the senescence program leads to detrimental effects in tissue homeostasis and function (Kwon et al., 2017).

The ability to engage the cell cycle program has always been considered a prerequisite for developing cellular senescence. However, several studies have demonstrated that even terminally differentiated neurons may acquire a senescence‐like state during physiological aging and under pathological conditions (Sapieha & Mallette, 2018). Accordingly, *in vitro* and *in vivo* studies have shown that differentiated neurons display a consistent increase in senescence markers, including senescence‐associated β‐galactosidase (SA‐β gal) activity, inhibition of cell cycle regulatory genes, lipofuscin accumulation, p21^CIP1/WAF1^‐dependent mitochondrial dysfunction, and activation of the senescence‐associated secretory phenotype (SASP) genes (Ishikawa & Ishikawa, 2020). Accumulating evidence has shown that dysregulation of the autophagic process contributes to senescence acquisition (Kwon et al., 2017). Together with the ubiquitin‐proteasome system, autophagy is one of two major protein degradation systems inside the cell. Autophagic flux is responsible for the degradation of protein and organelles through lysosomes in response to various stimuli, including nutrient deprivation, growth factor withdrawal, hypoxia, and oxidative stress (Stavoe & Holzbaur, 2019). Recent studies in nematodes and mammals have shown that the aging brain displays reduced levels of autophagic activity, thus losing the ability to prevent waste accumulation (Hansen et al., 2018). Remarkably, positive regulators of autophagy reduce brain aging and extend the animal lifespan (Nakamura & Yoshimori, 2018).

Among the numerous genes associated with aging, recent studies indicate the repressor element‐1 silencing transcription factor (REST)/neuron‐restrictive silencer factor (NRSF) as major protective factor (Lu et al., 2014; Zullo et al., 2019). REST is a member of the Kruppel‐type zinc finger transcription factor family and is essential for proper neuronal differentiation, axonal growth, neurotransmitter release, and control of excitability (Hwang & Zukin, 2018).

During physiological aging, REST accumulates and preserves neuronal function via the repression of apoptotic and oxidative stress genes (Lu et al., 2014). Moreover, growing evidence based on data from patients with Alzheimer's disease (AD) or Parkinson's disease (PD), and animal models thereof has indicated the decreased expression and activity of REST under pathological conditions (Ashton et al., 2017; Huang et al., 2019; Lu et al., 2014; Meyer et al., 2019; Zullo et al., 2019). Notably, impairments of the autophagic flux have been described in a large group of neurodegenerative disorders, including AD and PD (Nixon, 2013). However, no studies providing a causal relationship between REST, cellular senescence, and autophagy have been conducted to date.

Here, we show that REST affects the senescence program in cortical neurons. Specifically, the genetic reduction of REST leads to the expression of a senescence phenotype through the upregulation of inhibitor of cyclin‐dependent kinases p21 (CDKN1A) and the reduction of cell viability. We found that REST directly contributes to cellular senescence by interacting with the autophagic machinery and that the specific inhibition of REST impairs autophagy and mitochondrial function. Finally, induction of autophagy in neurons silenced for REST expression rescues p21 expression, and mitochondrial function, thus ameliorating the senescence phenotype.

## 2 RESULTS

### 2.1 Loss of REST expression results in cell cycle exit and decrease viability of cortical neurons

To investigate the role of REST in the acquisition of senescence markers, we employed postnatal primary cortical neurons derived from REST ^GTi/ GTi^ mice, a model bearing a conditional gene trap (GTi) cassette in an intron of the endogenous REST gene (Nechiporuk et al., 2016). REST depletion was induced by transduction of lentiviral particles encoding for Cre recombinase to terminate REST transcription (herein referred to as Cre‐REST). As a control, we used transduction with an inactive form of Cre recombinase (herein referred to as ΔCre‐REST).

**FIGURE 1:**
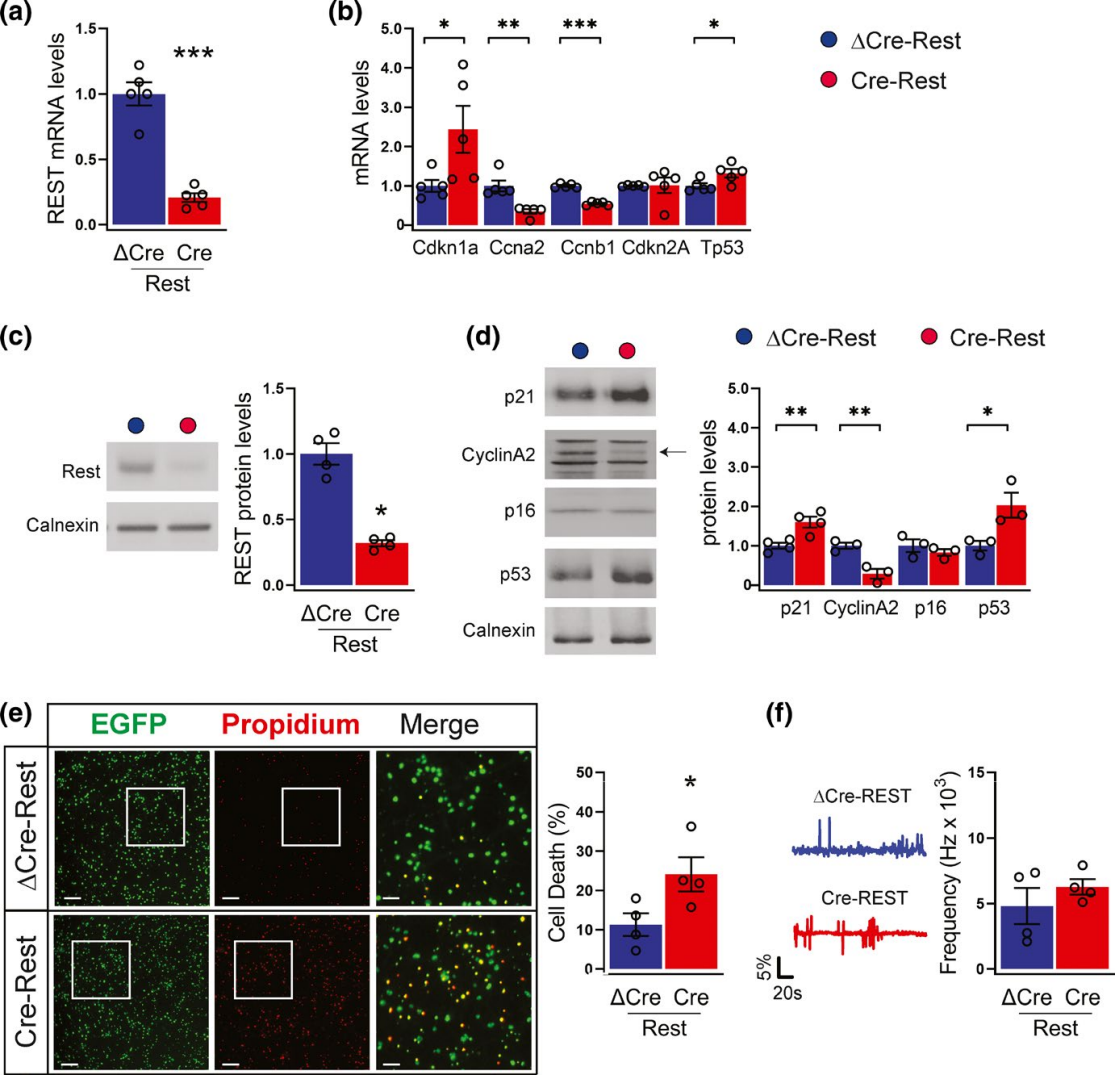
REST depletion alters the expression of cell cycle regulators (a,b) qRT‐PCR was used to measure mRNA levels of REST (a), cyclin‐dependent kinase inhibitor 1A (Cdkn1a), cyclin A2 (Ccna2), cyclin B1 (Ccnb1), cyclin‐dependent kinase inhibitor 2A (Cdkn2A) and p53 (Tp53) (b) in ΔCre‐REST (blue bars) and Cre‐REST (red bars) neurons; *n* = 5 independent preparations. Gapdh, Actin, and GusB were used as control housekeeping genes. (c,d) Western blotting analysis was used to confirm REST depletion (c) and measure the protein levels of p21, cyclin A2, p16, and p53 (d) in ΔCre‐REST and Cre‐REST neurons. An arrow points to the specific band for cyclin A2. Calnexin was used as loading control. Graphs show means ±sem of n = 3–5 independent preparations. (e) Cell death was evaluated by fluorescence microscopy of neurons transduced to express nuclear EGFP and double stained with propidium iodide (PI). Transduced neurons are EGFP tagged. The percentages of PI^+^ cells with respect to the total number of EGFP^+^ cells were calculated for each experimental group. Scale bar, 50 µm. Graphs show means ± sem from *n* = 4 independent experiments. A total number of 12 coverslips (5 fields/coverslip) were imaged per condition. (f) Representative traces (left) and frequency of calcium oscillations (right) in ΔCre‐REST and Cre‐REST transduced cortical neurons. Recordings were performed at 14 DIV. Peaks with at least 2% increase with respect to the baseline were considered. Graphs show means ± sem from *n* = 4 independent experiments. *p <0.05; **p < 0.01; ***p < 0.001; unpaired two‐tailed Student's *t* test (a,d,e); Mann–Whitney *U* test (b,c,f)

Quantitative real‐time PCR (qRT‐PCR) and western blotting analysis confirmed the effective downregulation of REST mRNA and protein, respectively (Figure 1a,c). The low residual expression of REST that was still detected is compatible with the observed viral transduction rate of ≈80%–90%.

We first assessed the expression of cell cycle regulatory and DNA damage genes. Compared with ΔCre‐REST controls, Cre‐REST neurons showed significantly higher expression of the cyclin‐dependent kinase (CDK) inhibitor p21 (Cdkn1a), largely recognized as a pro‐senescence factor and REST target gene (Nechiporuk et al., 2016; Zhang et al., 2017), whereas cyclin A2 (Ccna2) and cyclin B1 (Ccnb1) were downregulated (Figure 1b). p21 is one of the most important targets of p53 transcriptional activity in senescent cells (Qian & Chen, 2013). Consistent with the activation of p21 signaling, REST deficiency significantly increased p53 mRNA levels, while p16 (Cdkn2a) expression showed no significant change (Figure 1b). By checking the protein levels by western blotting analysis, we found that changes in protein expression were largely consistent with the changes in mRNA levels (Figure 1d). These results are in line with previous studies reporting that deletion of REST is associated with cell cycle exit in both neural progenitors and cardiomyocytes (Nechiporuk et al., 2016; D. Zhang et al., 2017). In addition, the possibility that the observed results are due to the presence of astrocytes can be ruled out, as the cultures express a minimum amount of the astrocyte‐specific marker glial fibrillary acidic protein (GFAP) under our experimental conditions (Figure S1,a,b). We next monitored the cell death rate of Cre‐REST neurons by quantifying incorporation of propidium iodide (PI). We found that REST deficiency negatively affected neuronal survival, with a twofold increase of dead cells with respect to ΔCre‐REST‐transduced controls (Figure 1e). On the contrary, the viability of non‐transduced cells in Cre‐REST‐transduced samples was not significantly affected with respect to control ΔCre‐REST‐transduced cultures (Figure S2), excluding that Cre‐REST neurons induce cell death in surrounding bystander cells over the investigated *in vitro* period. In spite of the alterations in neuronal survival, Cre‐REST neurons did not display differences in network excitability, as evaluated by monitoring spontaneous calcium oscillations by Fura‐2AM live imaging (Figure 1f), as previously reported (Buffolo et al., 2021).

### 2.2 REST downregulation in cortical neurons promotes a senescence phenotype

**FIGURE 2:**
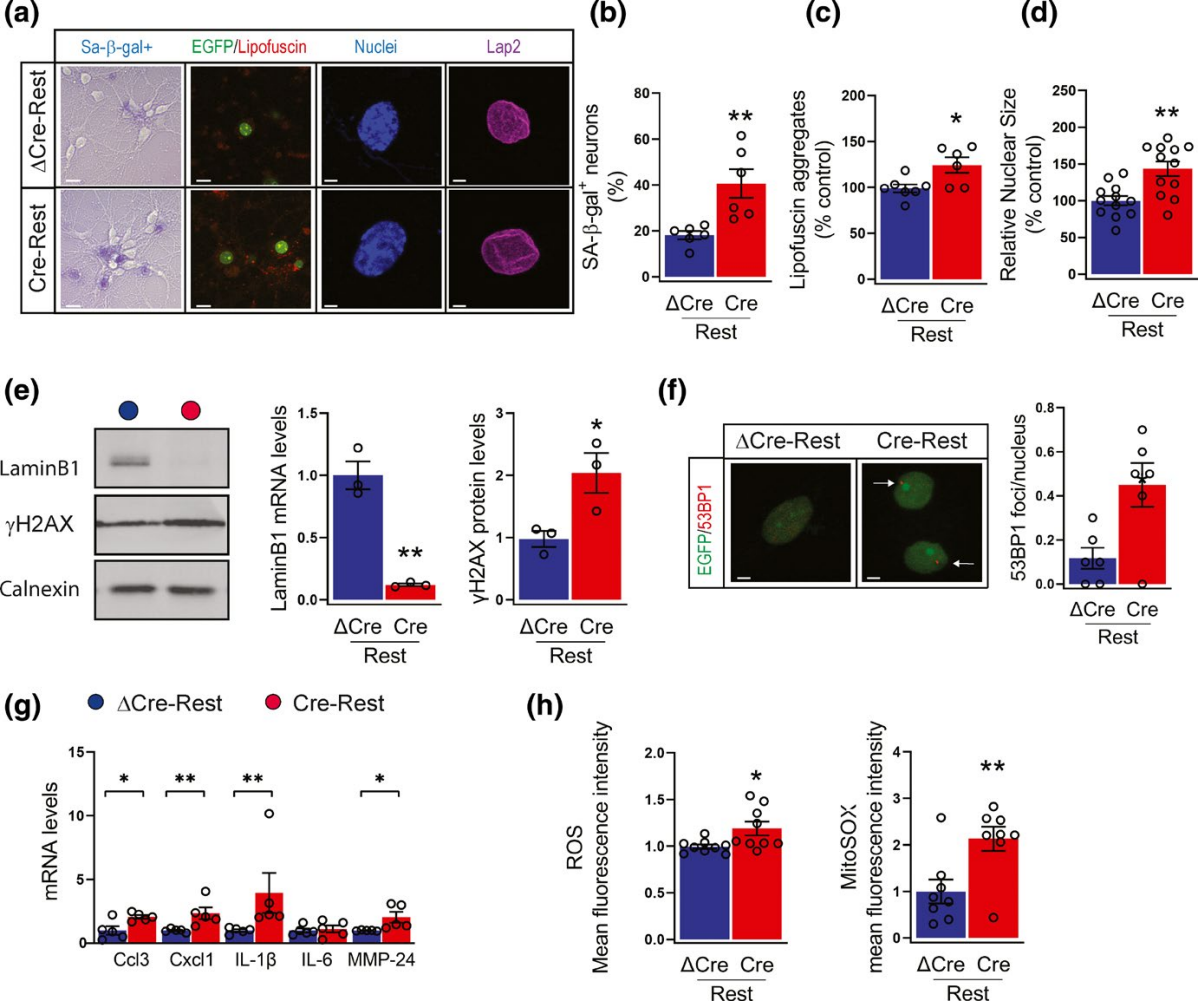
REST inhibition triggers a neuronal senescence phenotype (a) Representative images of (*from left to right*): senescent cells stained with 5‐Br‐4‐Cl‐3‐indolyl‐b‐D‐galactopyranoside (β‐gal; violet); autofluorescent lipofuscin aggregates (red); cell nuclei stained with Hoechst 33342 (blue); nuclear blebs stained with Lap2 (purple) in ΔCre‐REST and Cre‐REST neurons. Scale bar, 5 µm. (b‐d) quantification of β‐gal staining (b; *n* = 6 independent preparations), lipofuscin aggregates quantified by measuring their pixel area/field (c; *n* = 6 independent preparations); nuclear size (d; *n* = 12 replicates from 6 independent preparations) in ΔCre‐REST (blue bar) and Cre‐REST (red bar) neurons. (e) Representative images (*left*) and quantitative western blotting (*right*) of lamin B1 and γH2AX in ΔCre‐REST and Cre‐REST neurons. Calnexin was used as loading control. *n* = 3–4 independent preparations. (f) Representative images (*left*) and quantification (*right*) of 53BP1 foci in ΔCre‐REST and Cre‐REST neurons. The arrows point to 53BP1 foci. Scale bar, 5 µm. *n* = 12 from 6 independent preparations. (g) qRT‐PCR analysis of mRNA levels of Ccl3, Cxcl1, IL‐1β, IL‐6, MMP‐24 in ΔCre‐REST and Cre‐REST neurons Gapdh, Actin, and GusB were used as control housekeeping genes. *n* = 5 independent preparations. (h) Quantification of cellular ROS (*left*) and mitochondrial ROS (*right*) levels by Cell Rox and Mitosox labeling, respectively. *n* = 8–9 replicates from 3 independent experiments. Graphs show means ± sem *p < 0.05; **p < 0.01; ***p < 0.001; unpaired two‐tailed Student's *t* test (a,b,c,d,e,f,h); Mann–Whitney *U* test (g)

Given the relationship between the senescence and cell cycle arrest, we analyzed several features of the senescence state in Cre‐REST cortical neurons at 14 DIV. First, we observed a robust increase in acidic lysosomal (SA‐β‐gal) activity, a key senescence marker, as compared with ΔCre‐REST controls (Figure 2a,b). REST‐deficient neurons also displayed a significant increase in the intracellular accumulation of granules of lipofuscin, a non‐degradable aggregate of oxidized proteins and lipids that is also largely recognized as senescence marker (Figure 2a,c).

Cellular senescence is associated with alterations in nuclear shape and lamina; in line with this, we found that REST‐depleted neurons were characterized by a significant increase in nucleus diameter associated with reduced lamin B1 expression (Figure 2a,d,e). Although earlier studies suggested that nuclear membrane alterations may occur at sites of nuclear blebs (Coffinier et al., 2011), both ΔCre‐ and Cre‐REST transduced neurons displayed very rare nuclear blebs labeled by the inner nuclear membrane marker LAP2β (lamina‐associated polypeptide 2, β isoform) (Figure 2a). However, we suspected that lamina alterations could lead to DNA damage. Indeed, the phosphorylated form of H2AX histone (γ‐H2AX), an early indicator of DNA double‐strand breaks, was increased in Cre‐REST neurons compared with ΔCre‐REST controls (Figure 2e). Next, we assessed the number of tumor protein p53 binding protein (53BP1) foci, a mediator of DNA repair. Similar to γH2AX, we also observed an increased number of 53BP1 foci in Cre‐REST neurons (Figure 2f).

Consistent with these observations, REST‐depleted cultures exhibited upregulation of pro‐inflammatory chemokines, as chemokine (C‐C motif) ligand 3 (Ccl3) and chemokine (C‐X‐C motif) ligand 1(Cxcl1), interleukin IL‐1β (but not IL‐6), and matrix metalloprotease 24 (MMP‐24), reflecting the activation of the senescence‐associated secretory phenotype (SASP) (Figure 2g). Notably, alterations in the expression of SASP gene, senescence and DNA damage markers preceded those in cell death, as they were detected as early as 11 DIV, when the viability of Cre‐REST transduced neurons was still preserved (Figure S3a‐d).

In addition, REST‐depleted neurons were characterized by increased levels of reactive oxygen species (ROS) and especially superoxide, a mitochondria‐specific ROS class (Figure 2h).

Collectively, these data show that REST depletion in primary neurons negatively regulates cell cycle regulatory genes, precociously triggers the senescence program.

### 2.3 The senescence phenotype induced by REST downregulation in cortical neurons is associated with mitochondrial dysfunction

**FIGURE 3:**
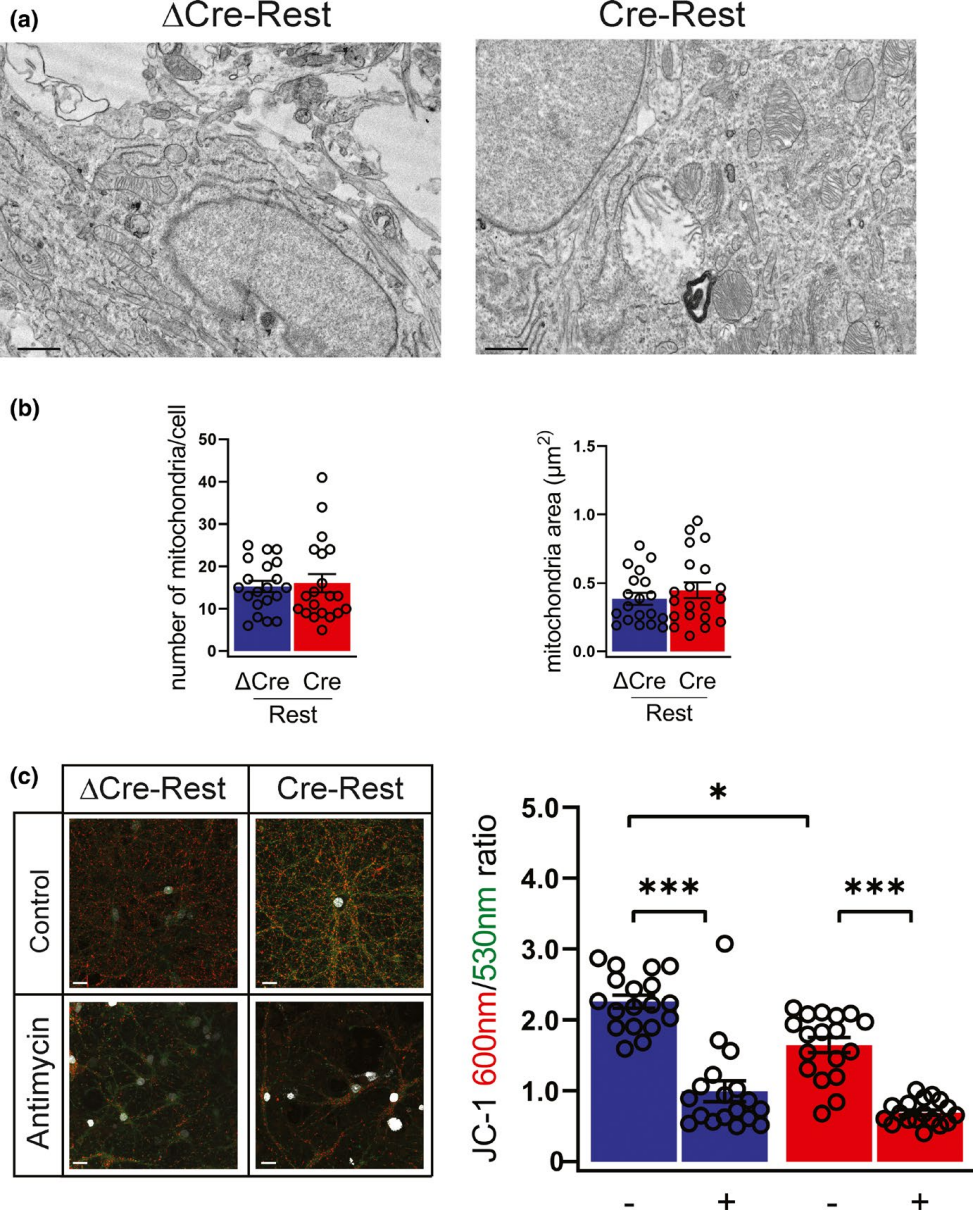
REST‐defective neurons have impaired mitochondrial membrane potential in the absence of morphological alterations (a,b) Representative micrographs (a) and quantification (b) of the number (*left panel*) and area (*right panel*) of mitochondria in ΔCre‐ and Cre‐transduced neurons. *n* = 20 neurons from 3 independent preparations. Scale bar, 0.5 μm. (c) Representative images (*left*) and quantification (*right*) of JC‐1 staining in ΔCre‐REST (blue bar) and Cre‐REST (red bar) neurons in the presence or absence of antimycin (40 mM, 1 h). JC‐1 accumulates in mitochondria in a ΔΨ_m_‐dependent manner; it exists as monomer at low concentrations (emission, 530 nm) and forms aggregates at higher concentrations (emission, 600 nm). Antimycin A (40 mM, 1 h), a complex III inhibitor, leads to a rapid breakdown of ΔΨ_m_, confirming the dynamic assay properties. Scale bar, 5 µm. *n* = 18 coverslips from 4 independent preparations. Graphs show means ± sem. **p < 0.01; ***p < 0.001; Kruskal–Wallis/ Dunn's tests

Emerging evidence indicate that mitochondria play a key role in the development of senescence, particularly of the pro‐inflammatory SASP. To understand whether the decreased REST expression resulted in mitochondrial dysfunction, we performed an ultrastructural analysis of mitochondria in Cre‐REST‐ and ΔCre‐REST transduced primary neurons (Figure 3a). Morphometric analysis of the micrographs did not reveal any significant mitochondrial abnormality, both in terms of number and mean area of neuronal mitochondria in Cre‐REST neurons as compared with ΔCre‐REST controls (Figure 3b). Next, we analyzed the possibility of a purely functional mitochondrial dysfunction. To assess this, we labeled neurons with the JC‐1 dye, known to monitor mitochondrial potential independently of changes in mitochondrial shape/size or density. Interestingly, loss of REST drives significantly decreased mitochondrial membrane potential, as shown by JC‐1 labeling (Figure 3c). These data demonstrate that a mitochondrial dysfunction is associated with the activation of the senescence program.

### 2.4 REST‐depleted neurons display impaired autophagy

An autophagic impairment has been associated with cellular senescence (Rubinsztein et al., 2011). Therefore, we asked whether REST depletion could affect the occurrence of autophagic process. We started by analyzing the expression of the microtubule‐associated protein 1A/1B‐light chain 3 (LC3), a well‐known marker of autophagosomes (Klionsky et al., 2016).

**FIGURE 4:**
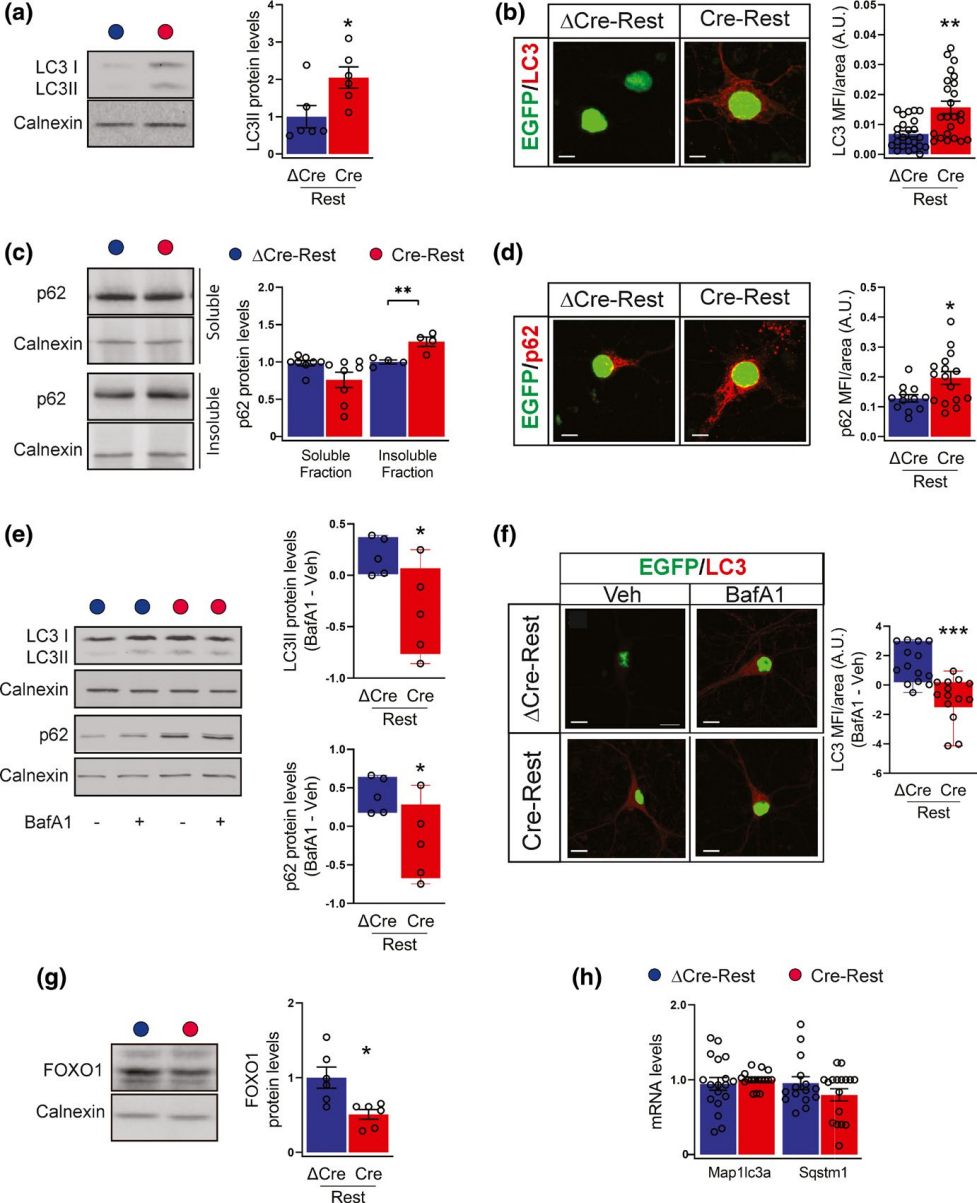
REST deficiency negatively affects the autophagic flux (a) Representative blots (*left*) and quantitative analysis (*right*) of LC3II (lower band) in ΔCre‐REST (blue bar) and Cre‐REST (red bar) transduced neurons. Calnexin was used as loading control. Results are expressed as LC3II/calnexin ratio. *n* = 6 independent preparations. (b) Representative images (*left*) and quantification (*right*) of ΔCre‐ and Cre‐transduced neurons stained for LC3 (red). Transduced neurons are EGFP‐tagged. *n* = 27 coverslips (5 fields/coverslip) from 3 independent preparations. Scale bars, 20 μm. (c) Representative blot (*left*) and quantitative analysis (*right*) of detergent‐soluble and ‐insoluble fractions from ΔCre‐ and Cre‐transduced neurons immunoblotted for p62. Calnexin was used as loading control. *n* = 4–8 from 3 independent preparations. (d) Representative images and quantification of ΔCre‐ and Cre‐transduced neurons stained for p62 (red). Transduced neurons are EGFP‐tagged. *n* = 12–16 coverslips from 3 independent preparations. Scale bars, 20 μm. (e) Representative blots (*left*) and quantitative analysis (*right*) of LC3II (lower band) and p62 in ΔCre‐ and Cre‐transduced neurons treated with either vehicle or BafA1 (300 nM, 8 h). Calnexin was used as loading control. Results are expressed as difference between BafA1 and Veh condition. *n* = 5 independent preparations. (f) Representative images (*left*) and quantification (*right*) of ΔCre‐ and Cre‐transduced neurons treated with either vehicle or BafA1 (300 nM, 8 h) and stained for LC3 (red). Results are expressed as differences between BafA1 and Veh condition. Transduced neurons are EGFP‐tagged. *n* = 14–30 coverslips from 3 independent preparations. Scale bars, 20 μm. (g) Representative blots (*left*) and quantitative analysis (*right*) of FOXO1 in ΔCre‐ and Cre‐transduced neurons. Calnexin was used as loading control. Results are expressed as FOXO1/calnexin ratio. *n* = 5 independent preparations. (h) The mRNA levels of Map1lc3a (encoding for LC3) and Sqstm1 (encoding for p62) were quantified by qRT‐PCR in ΔCre‐ and Cre‐transduced neurons. Gapdh, Actin and GusB were used as control housekeeping genes. n = 4 independent preparations. *p < 0.05; **p < 0.01; ***p < 0.001; unpaired two‐tailed Student's *t* test (a,c,e,g) and Mann–Whitney *U* test (b,d,f)

REST‐depleted neurons express higher levels of the lipidated form of LC3 (LC3II) generated during autophagosome formation, as shown by western blotting analysis of cell lysates and immunofluorescence (Figure 4a,b). We also monitored the levels of the selective autophagy receptor protein sequestosome 1/p62 (SQSTM1/p62), an adaptor protein responsible for recognizing and loading cargo substrates into autophagosomes (Klionsky et al., 2016). We observed a significant accumulation of p62 in the detergent‐insoluble fraction of REST‐depleted neurons, whereas no difference was present in the detergent‐soluble fraction (Figure 4c). The p62 accumulation was also confirmed by quantitative immunofluorescence which showed that REST‐depleted neurons were characterized by an intense p62 immunoreactivity in the cytosol, as compared with ΔCre‐REST transduced controls (Figure 4d).

These findings suggest that autophagic activity is depressed in Cre‐REST cultures. To unambiguously demonstrate the impairment of autophagic process, we used the autophagy‐flux inhibitor bafilomycin A1 (BafA1), which prevents lysosome degradation and increases LC3 and p62 expression exclusively when autophagy is active. While, as expected, the treatment induced a significant accumulation of LC3II and p62 in ΔCre‐REST control cultures, BafA1 was totally ineffective in increasing the LC3II and p62 expression in REST‐depleted neurons (Figure 4e,f).

Accordingly, the expression of forkhead box O1 (FOXO1), a transcription factor positively associated with the induction of autophagy and stress resistance (Wang et al., 2016), was impaired in Cre‐REST cultures (Figure 4g). Finally, no differences between the two experimental groups were observed in LC3 and p62 transcript expression (*Map1lc3a* and *Sqstm1*, respectively) (Figure 4h), ruling out the possibility that the increased LC3 and p62 levels may depend on a direct activation of gene transcription.

### 2.5 Ultrastructural and transcriptional analysis reveals that REST‐depleted neurons have a strongly impaired autophagic flux

**FIGURE 5:**
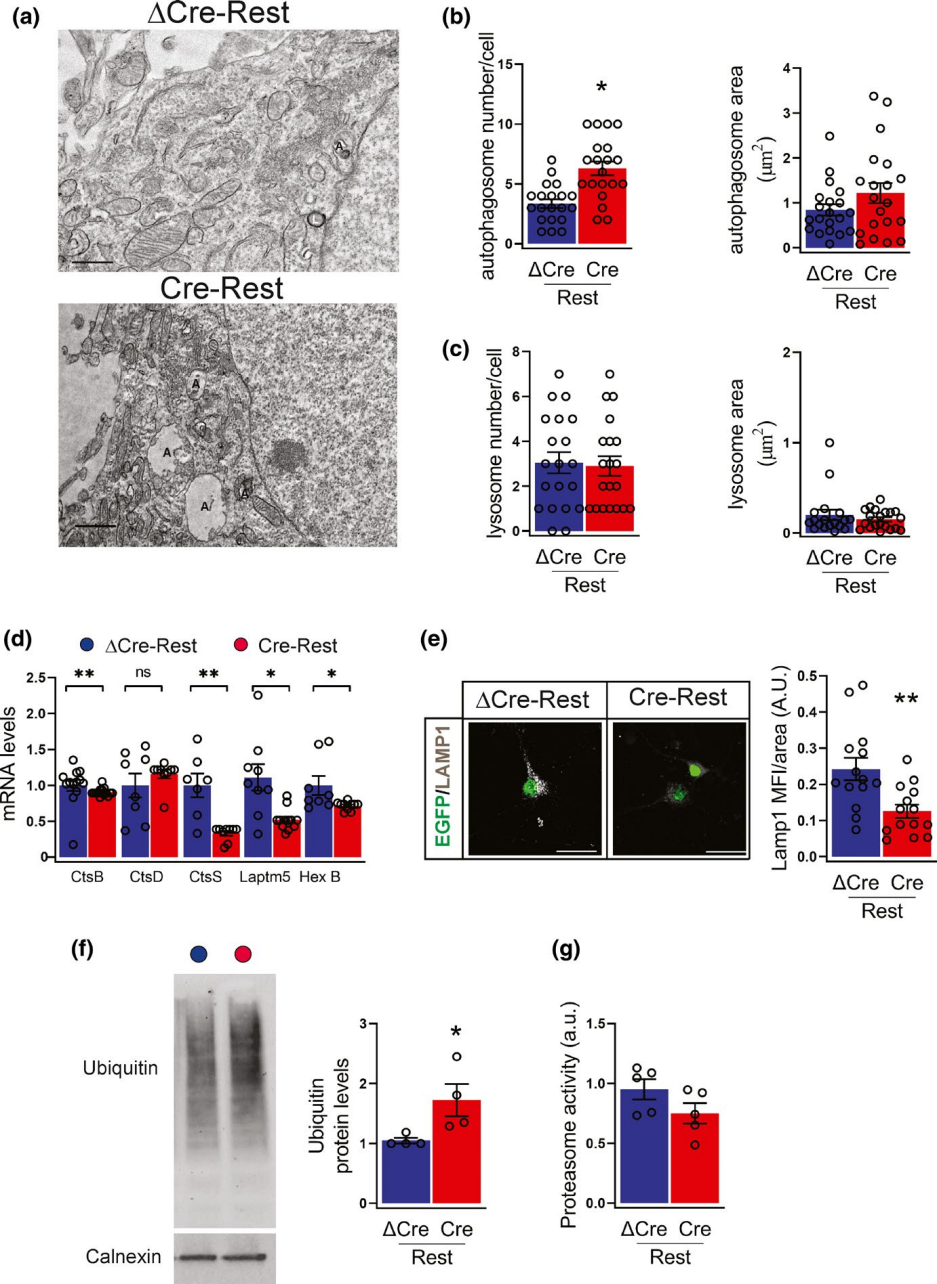
REST‐depleted neurons have an impaired autophagic flux (a) Representative micrographs of ΔCre‐ and Cre‐transduced neurons showing the presence of autophagosomes (indicated with an “A”) and lysosomes (marked with an “L”). Scale bar, 0.5 μm. (b,c) Quantification of the density (*left*) and area (*right*) of autophagosomes (b) and lysosomes (c) in ΔCre‐ (blue bars) and Cre‐(red bars) transduced neurons. *n* = 20 neurons from 3 independent preparations. (d) The mRNA levels of cathepsin B (CtsB), cathepsin D (CtsD), cathepsin S (CtsS), lysosomal‐associated protein transmembrane 5 (Laptm5), hexosaminidase subunit beta (HexB) were quantified by qRT‐PCR in ΔCre‐ and Cre‐transduced neurons. Gapdh, Actin, and GusB were used as control housekeeping genes. *n* = 8–15 replicates from 4 independent preparations. (e) Representative images (*left*) and quantification (*right*) of ΔCre‐ and Cre‐transduced neurons stained for Lamp1 (white). Transduced neurons are EGFP tagged. *n* = 14 coverslips from 4 independent preparations. Scale bars, 20 μm. (f) Representative western blot (*left*) and quantification (*right*) of protein ubiquitination in ΔCre‐ and Cre‐transduced neurons. Ubiquitinated proteins were detected with anti‐ubiquitin antibody, using calnexin as loading control. *n* = 4 independent preparations. (g) Proteasome activity in ΔCre‐ and Cre‐transduced neurons. *n* = 4 independent preparations. Graphs show means ± sem. *p < 0.05; **p < 0.01; unpaired two‐tailed Student's *t* test for (b,e,f) and Mann–Whitney *U*‐test for (d)

We next performed an ultrastructural analysis by conventional fixation‐embedding procedures to identify autophagosomes and lysosomes based on their size and morphology. REST‐depleted neurons showed a significant twofold accumulation of autophagosomes with respect to ΔCre‐REST controls, with no changes in their average area (Figure 5a,b). On the opposite, both the number and average area of lysosomes were not altered by REST deletion (Figure 5a,c).

SA‐β‐gal activity is a marker of altered function and enlargement of lysosomal compartments (Figure 2a,b). To test the possibility that lysosomal function was altered by REST depletion in the absence of overt morphological changes, we performed qRT‐PCR analysis of core lysosome genes. Interestingly, we found that REST depletion caused the transcriptional repression of several lysosomal functional genes, including cathepsin B (CtsB), cathepsin S (CtsS), lysosomal‐associated protein transmembrane 5 (Laptm5), and hexosaminidase subunit beta (HexB) (Figure 5d). Consistently, the density of Lamp1 lysosomal puncta in EGFP‐tagged Cre‐REST‐transduced neurons was significantly decreased with respect to ΔCre‐REST control neurons (Figure 5e). Similar to what observed with mitochondria, these results indicate that, regardless the apparently normal lysosome distribution and number, the lysosomal function is significantly perturbed by REST deletion.

A long array of studies have shown that the crosstalk between autophagy and the proteasomal system and that autophagic dysregulation affects the ubiquitin proteasomal system (UPS; Dikic, 2017). To further clarify this aspect, we analyzed the level of ubiquitinated proteins and the proteasome activity in total protein lysates. The amount of ubiquitinated proteins was increased by about twofold in REST‐depleted neurons compared with control neurons; however no significant change in proteasome activity was detected in both experimental groups (Figure 5f,g).

Taken together, these results strongly suggest that REST deficiency leads to loss of proteostasis due to impairment of autophagic flux rather than of UPS.

### 2.6 Restoration of autophagy in REST‐depleted neurons reverts the senescence phenotype

**FIGURE 6:**
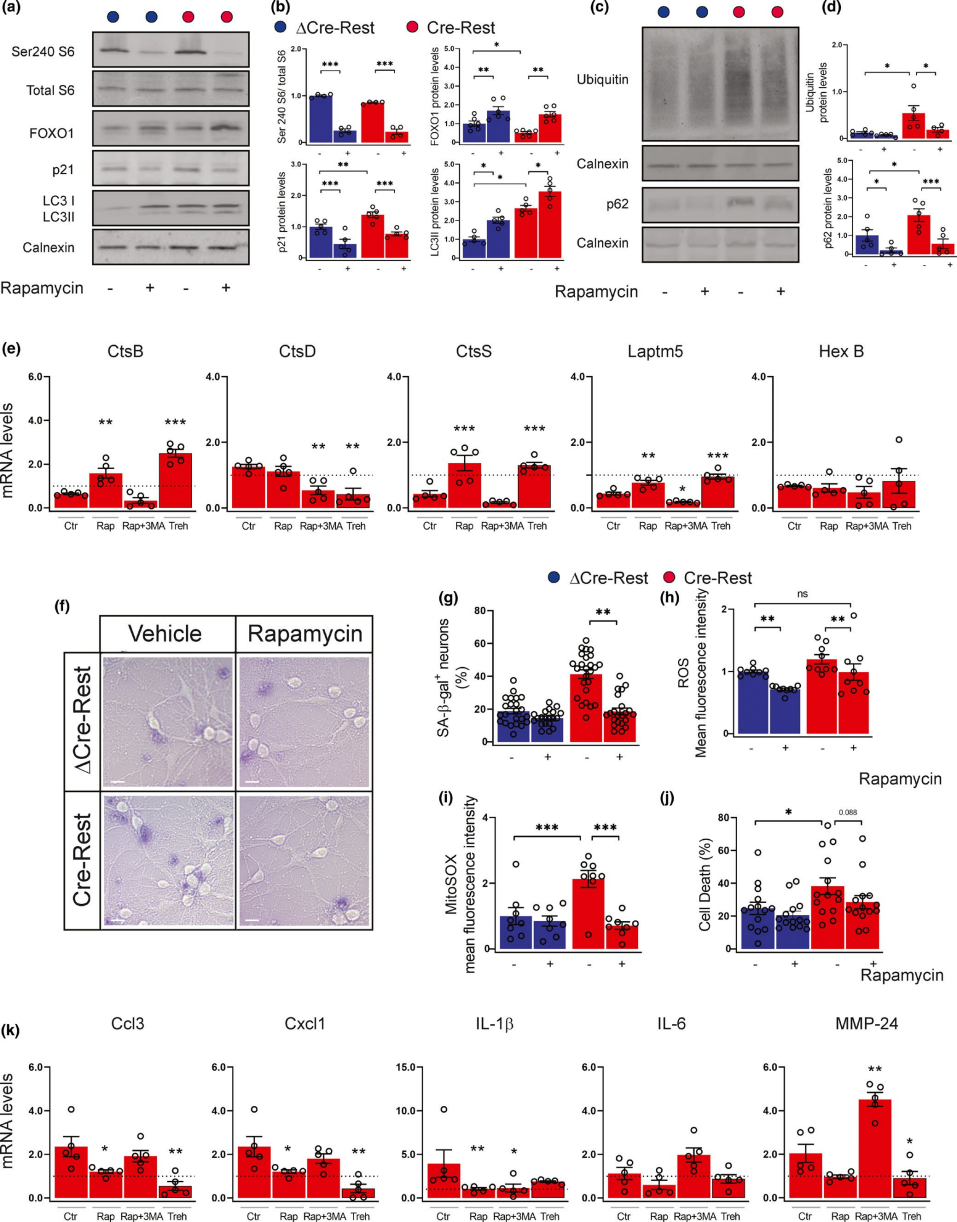
Rapamycin treatment ameliorates REST‐dependent senescence phenotype (a,b) Representative western blotting (a) and quantification (b) of phosphorylated and total S6, FOXO1, p21 and LC3I/LC3II in ΔCre‐REST and Cre‐REST transduced neurons treated with either vehicle (‐) or 30 nM rapamycin (+) for 72 h. Calnexin was used as loading control. *n* = 4–6 independent preparations. (c,d) Representative western blotting (c) and quantification (d) of ubiquitin and p62 levels. Calnexin was used as loading control. *n* = 4–6 independent preparations. (e) The mRNA levels of CtsB, CtsD, CtsS, Laptm5, and HexB were quantified by qRT‐PCR in Cre‐transduced neurons incubated either with vehicle (Ctr), rapamycin (Rap), rapamycin plus 3‐MA (Rap +3‐MA) or trehalose for 72 h. Gapdh, Actin, and GusB were used as control housekeeping genes. *n* = 5 independent preparations. The dotted line indicates the control condition (vehicle‐treated ΔCre‐REST neurons). (f) Bright light representative images of ΔCre‐ and Cre‐transduced neurons treated with either vehicle (‐) or 30 nM rapamycin (+) for 72 h and stained with SA‐β‐gal to label senescent cells. Scale bar, 5 µm. (g) Quantification of the number of SA‐β‐gal‐stained ΔCre‐ (blue bars) and Cre‐ (red bars) transduced neurons treated with either vehicle (‐) or 30 nM rapamycin (+) for 72 h. *n* = 20–25 coverslips (5 fields/coverslip) from 3 independent preparations. (h,i) Cellular ROS levels quantified by Cell Rox labeling (h) and mitochondrial ROS quantified by MitoSOX labeling (i) in ΔCre‐ and Cre‐transduced neurons treated with either vehicle (‐) or 30 nM rapamycin (+) for 72 h. *n* = 9 replicates from 3 independent preparations. (j) Cell death evaluated under the same experimental conditions by incorporation of propidium iodide. *n* = 14 coverslips (5 fields/coverslip) from 3 independent preparations. (k) The mRNA levels of Ccl3, Cxcl1, IL‐1β, IL‐6, MMP‐24 were quantified by qRT‐PCR in Cre‐transduced neurons incubated either with vehicle (Ctr), rapamycin (Rap), rapamycin plus 3‐MA (Rap +3‐MA) or trehalose for 72 h. The dotted line indicates the control condition (vehicle‐treated ΔCre‐REST neurons). Gapdh, Actin, and GusB were used as control housekeeping genes. n = 5 independent preparations. Graphs show means ± sem. *p <0.05; **p < 0.01; ***p < 0.001; ns: not significant. Two‐way ANOVA/Bonferroni's tests (b,d,g,h,i, j), one‐way ANOVA/Dunnett's tests versus vehicle (e,k)

It widely demonstrated that the loss of proteostasis contributes to cellular senescence in several experimental models (Ishikawa & Ishikawa, 2020). Thus, we investigated whether restoring autophagy could reverse the expression of the senescence program triggered by REST depletion in neurons. To test this hypothesis, we treated neurons for 72 h with either vehicle or rapamycin, a specific mTORC1 inhibitor and a strong inducer of autophagy. Rapamycin treatment was effective in both REST‐depleted neurons and ΔCre‐REST control neurons as shown by the marked reduction of the phosphorylation state of ribosomal protein S6 (Figure 6a,b). Rapamycin treatment activated autophagy as measured by the increased LC3II expression, suggesting that REST‐depleted neurons are still capable of engaging autophagy (Figure 6a,b). Consistent with this finding, FOXO1 and p21 (Figure 6a,b), as well as ubiquitin and p62 levels (Figure 6c,d) were also restored in Cre‐REST neurons to values comparable with ΔCre‐REST neurons by rapamycin treatment. Ultrastructural analysis confirmed the activation of autophagic flux, as demonstrated by the increased density of autophagosomes in both rapamycin‐treated groups (Figure S4).

The restoration of autophagy was also able to restore the expression of the lysosomal genes CtsB, CtsS and Laptm5 in REST‐depleted neurons to the levels observed under basal conditions in ΔCre‐REST control neurons (Figure 6e). To clarify whether the effects of rapamycin treatment were mainly dependent on its ability to regulate autophagy, we analyzed the same gene panel in response to the combined treatment with rapamycin and the autophagy inhibitor 3‐methyladenine (3‐MA). We found that the effect of rapamycin on CtsB, CtsS, and Laptm5 was reversed when Cre‐REST cells were simultaneously treated with 3‐MA, suggesting that the effects on lysosomal genes are blocked when autophagy is inhibited (Figure 6e; Figure S5a). To further investigate whether the effects of rapamycin were autophagy‐dependent, neurons were treated with trehalose, a disaccharide that is able to induce autophagy via an mTOR‐independent pathway. Notably, the effects of trehalose on lysosomal genes were fully comparable to those of rapamycin, demonstrating that the observed changes are independent of the mTOR pathway (Figure 6e; Figure S5a).

We demonstrated that the induction of autophagy reverted the neuronal senescence program initiated by REST depletion. Rapamycin not only decreased the activation of p21 in REST‐depleted neurons (Figure 6a,b), but also significantly decreased the number of SA‐β‐gal‐positive neurons (Figure 6f,g) and restored the physiological ROS and mitochondrial superoxide to the levels observed under basal conditions in ΔCre‐REST control neurons (Figure 6h,i). However, rapamycin was not able to prevent cell death in Cre‐REST transduced neurons (Figure 6j). Interestingly, Cre‐REST neurons incubated with either rapamycin or trehalose showed a significant reduction in the expression of SASP genes compared with untreated Cre‐REST neurons, whereas rapamycin +3‐MA treated cells showed an expression level similar to the control (Figure 6k; Figure S5b). Taken together, these results show that restoration of autophagy is sufficient to counteract the senescence phenotype in REST‐deficient neurons.

## 3 DISCUSSION

The progression of aging depends on a series of sequential and interconnected events that include the induction of cellular senescence. A large body of research has shown that senescence can be triggered by a variety of stimuli and can vary in a cell type‐ and context‐dependent manner. Whether cells that underwent a terminal differentiation can acquire senescence features has been debated for a long time. Studies in different species have described the ability of neurons to engage a senescence program, characterized by DNA damage, high SA‐β‐gal activity and expression of pro‐inflammatory molecules that collectively are typical hallmarks of senescence (Jurk et al., 2012).

Among the numerous players implicated in the aging process, REST signaling has recently attracted a large attention (Lu et al., 2014; Yoshizaki et al., 2021; Zullo et al., 2019). REST upregulation has been described in human brain during physiological aging. REST contributes to reduce the overall neural excitation, apoptosis, and oxidative stress (Lu et al., 2014; Pecoraro‐Bisogni et al., 2018; Pozzi et al., 2013; Zullo et al., 2019). Here, we propose a novel mechanism to explain the role of REST in aging that involves an interaction between the senescence program and the autophagy. Our results indicate that REST deficiency promotes the expression of several senescence markers, including SA‐β‐gal, lipofuscin accumulation, enlargement of the nucleus and SASP gene expression. Moreover, primary neurons devoid of REST expression showed higher level of cell death, confirming the protective role of REST on neuronal survival (Lu et al., 2014; Zullo et al., 2019). Importantly, the appearance of the senescence phenotype preceded the apoptotic process, suggesting that the hallmarks of senescence are not induced by cellular toxicity in a non‐specific fashion.

The observation that the enhancement of senescence, long considered an anti‐apoptotic mechanism, increases the chance of cell death was somewhat unexpected. Numerous studies indicate that senescent cells develop greater resistance to oxidative stress and apoptosis. However, recent data have demonstrated the existence of an extremely complex interplay between senescence and apoptosis. It has been reported that specific cell populations are more susceptible to apoptosis during senescence and some hallmarks of late senescence mimic the apoptotic process (Childs et al., 2014). According to this view, we propose a model which identifies cell death as the consequence of a prolonged activation of senescence program.

Senescence is a cellular response activated by multiple stimuli, including DNA damage and oxidative stress. In this study, we found that REST deficiency induces double‐strand DNA in mouse cortical neurons, where it contributes to the activation of p53‐p21 axis which, in turn, negatively modulates cell cycle regulatory genes. Our study, in agreement with previous reports (Musi et al., 2018; Vazquez‐Villasenor et al., 2020; Zhang et al., 2020), argues that p21 overexpression prevents cell cycle re‐entry and sustains the activation of senescence program. This response can be protective in an early stage, but then contributes to toxicity by altering the cellular secretome. Senescence can occur as a consequence of excessive ROS production (Chapman et al., 2019). Consistently, we found that mitochondria from REST‐depleted neurons show a decreased mitochondrial membrane potential and an increased generation of ROS. Activation of signaling pathways regulating senescence, such as p21 and p53, have been shown to result in increased ROS. This suggest that ROS could act as signaling molecules enforcing the senescence phenotype.

We propose that REST contributes to cellular senescence by altering the autophagic process. A number of reports have described the complex response, involving the senescence program and cell protein turnover, which is mediated by the ubiquitin–proteasome system and the autophagy machinery. In the last decade, it has been shown that autophagy promotes oncogene‐induced senescence in several cellular models (Young et al., 2009). On the other hand, the impairment of autophagy promotes the senescence phenotype in fibroblasts, muscle cells, and neurons. These findings suggest that the role played by the autophagy process in cell activity and survival is dependent on the type of stimulus. While activation of oncogene‐induced autophagy may be a pro‐senescence factor, the basal autophagy can play a role as an anti‐senescence signal (Kwon et al., 2017).

In agreement with recent results obtained in dopaminergic neurons (Ryan et al., 2021), we show that REST deficiency contributes to cellular dysfunction by blocking autophagic protein clearance. We provide data for this blockade at both structural and functional levels. Evidence supporting the failure in the degradation of intracellular cargoes of REST‐depleted neurons include: (i) the elevated number of autophagosomes; (ii) the accumulation of autophagy substrate p62; (iii) the failure of BafA1 to further increase LC3II protein level; and (iv) the decreased expression of genes related to lysosomal function. Although autophagy and the ubiquitin–proteasome system are interacting systems that compensate each other, we found that only the autophagy machinery is impaired by REST depletion, whereas the ubiquitin–proteasome degradation is not affected. An important question is how REST deficiency impairs the autophagic flux. Autophagy is as a multistep process, consisting of initiation, expansion of the autophagosome membrane, maturation, and later lysosomal degradation. To identify at which step the autophagic flux is impaired in the absence of REST is essential to understand the sequence of events and identify a specific cellular pathway that might be specifically modulated. Although we were not able to precisely identify the stage responsible for defective autophagy, we demonstrated that the effect is, at least partially, dependent on FOXO1 activity. FOXO1 is a transcription factor, which promotes oxidative stress resistance, longevity and autophagy. Further studies will be required to clarify these aspects.

We showed the senescence phenotype can be reversed or at least delayed in cortical neurons when we restore the autophagic flux. Treatment of senescent cells with rapamycin, a positive inducer of autophagy, has been associated with decreased expression of senescence markers, reduction of oxidation and improvement of mitochondrial function in *in vitro* and *in vivo* models (Correia‐Melo et al., 2016, 2019). Consistently, we showed that the ability of rapamycin to rescue the autophagic flux is the key point to restore p21 expression, mitochondrial function, oxidative stress, and senescence in REST‐depleted neurons. This rescue does not involve secondary effects of mTOR inhibition, since trehalose, an alternative mTOR‐independent autophagy inducer, also decreases the expression of SASP genes and restores the expression of lysosomal genes in REST‐deficient neurons. The autophagy enhancing treatment, however, had only a modest effect in rescuing the increased rate of cell death observed in REST‐depleted neurons. This implies that alterations in the autophagic flux, even for relatively short periods during neuronal network development induces irreversible modifications leading to increased cell death.

Our data support the idea that the main features of aging, such as senescence and loss of proteostasis, are strictly interconnected. Although further experimental evidence is required, we hypothesize that enhancement of REST expression could represent a protective mechanism in response to aging‐associated changes, such as inflammatory conditions. Consistently, REST downregulation has been extensively reported in a number of neurodegenerative disorders associated with sustained immune response (Lu et al., 2014; Song et al., 2016; Zullo et al., 2019). In conclusion, we found that REST deficiency in primary neurons leads to the development of a senescence phenotype associated with an autophagic impairment. Our study identifies a new mechanistic link between REST and neuroprotection in primary neurons, suggesting promising molecular targets for age‐related neurodegenerative disorders.

## 4 EXPERIMENTAL PROCEDURES

### 4.1 Experimental animals

Primary postnatal cortical cultures were prepared from P0‐P1 pups from the previously described REST^GTi^ mice (Nechiporuk et al., 2016). All efforts were made to minimize suffering and reduce the number of animals used. All experiments were carried out in accordance with the guidelines established by the European Community Council (Directive 2010/63/EU of 22 September 2010) and were approved by the Italian Ministry of Health (authorization #558/2016‐PR).

### 4.2 Preparation and treatments of primary neurons

Pups were decapitated, and the cerebral cortex was removed and enzymatically dissociated. Cortical neurons were plated on poly‐L‐lysine‐coated (0.1 mg/ml) glass coverslips (Thermo‐Fischer Scientific) at a density of 40,000 cells/mL and maintained up to 14 days in vitro (DIV) at 37°C, 5% CO_2_, 95% humidity in a culture medium consisting of Neurobasal A, B‐27 (2%), GlutaMAX (1%), and penicillin/streptomycin (1%). All chemicals were purchased from Life Technologies/Thermo‐Fischer Scientific unless stated otherwise.

For the experiments aimed at studying the autophagic flux, 30 nM rapamycin (LC Laboratories, 72 h), 300 nM Bafilomycin A1 (BafA1, 8 h), 100 mM trehalose (72 h), 2 mM 3‐methyladenine (3‐MA, 72 h) (Sigma‐Aldrich, Milano, Italy) treatments were carried. As a control for both treatments, cultures were subjected to the same medium change with addition of an equivalent volume of vehicle.

The production of VSV‐pseudotyped third‐generation lentiviral particles was performed as previously described (Rocchi et al., 2019). pLenti‐PGK‐Cre‐EGFP or pLenti‐PGK‐ΔCre‐ EGFP plasmids were obtained as previously described (Kaeser et al., 2011). Primary neurons were infected at 7 DIV at a multiplicity of infection of 10. After 24 h, half of the medium was replaced with fresh medium.

### 4.3 RNA preparation and real‐time PCR analysis

Total RNA extraction and gene expression analysis were performed as previously described (Limongi et al., 2018). Gapdh, Actin, and Gusb were used as control housekeeping genes. The list of the used primers is provided in Table S1.

### 4.4 Biochemical analysis

For western blotting analysis, cells were harvested and lysed in RIPA buffer (50 mM Tris‐Cl pH 7.4, 150 mM NaCl, 2 mM EDTA, NP‐40 1%, and sodium dodecyl sulfate (SDS) 0.1%) supplemented with protease and phosphatase inhibitor cocktails (Roche Diagnostics). Cellular proteins were separated into detergent‐soluble and ‐insoluble fractions with the 2% Triton ×‐100 buffer [50 mM Tris (pH 8.0), 150 mM NaCl, 1 mM EDTA, 10% glycerol, 2% Triton ×‐100, a protein inhibitor mixture (Roche)]. The insoluble fractions were solubilized with 2% SDS, 20 mM HEPES, 5 mM EGTA. Protein concentration was determined using the BCA Protein Assay Kit (Thermo‐Fischer Scientific). Equal amounts of proteins were separated by SDS‐PAGE and blotted onto nitrocellulose membranes (GE Healthcare). The list of the used primary antibodies is provided in Table S2. Secondary antibodies were detected by chemiluminescence with the ChemiDoc imaging system (Biorad).

Proteasomal assay was performed as previously described (Rocchi et al., 2016). Briefly, cells were lysed using ice‐cold lysis buffer [50 mM HEPES, 5 mM EDTA, 150 mM NaCl, 2 mM ATP, 1% Triton×‐100], sonicated and centrifuged. Equal amounts (60 µg) of extracts were incubated for 2 h at 37°C in 70 µl of a solution containing 25 mM HEPES, 0.5 mM EDTA, 0.05% NP‐40, 0.001% SDS, and 0.5mM N‐succinyl‐Leu‐Leu‐Val‐Tyr‐7‐amido‐4‐methylcoumarin (Sigma). The absorption was recorded at 460 nm by excitation at 355 nm (Infinite F500 Microplate Reader, Tecan).

### 4.5 Transmission electron microscopy

Primary cortical neurons were prepared as previously described (Rocchi et al., 2019). Ultrathin sections (60–70 nm thick) were collected on 200‐mesh copper grids (EMS) and observed with a JEM‐1011 electron microscope (Jeol) operating at 100 kV using an ORIUS SC1000 CCD camera (Gatan). Images were acquired at 2500× magnification, and, for each experimental condition, 10–20 neuronal somas were reconstructed and processed offline by means of ImageJ software. Autophagosomes were identified as double‐membraned structures enclosing cytoplasmic components (Eskelinen, 2008), while lysosomes were identified conventionally as organelles with homogenous or sometimes coarsely granular matrix whose electron density is higher than that of the surrounding cytoplasm. For each neuron, the averaged area of its autophagosomes, lysosomes, or mitochondria was calculated.

### 4.6 Viability assay

Cells were incubated for 3 min at room temperature in extracellular medium (EM) (NaCl 135 mM, KCl 5.4 mM, MgCl_2_ 1 mM, CaCl_2_ 1.8 mM, glucose 10 mM, Hepes 5 mM, pH 7.4) containing 5 mg/mL propidium iodide (PI), and 3.3 μg/mL Hoechst 33342. After incubation, cells were washed once in EM and immediately imaged. The hardware configuration for the imaging experiments was based on a Nikon Eclipse 80i upright microscope (Nikon Instruments) equipped with an epifluorescence attachment and a digital camera Nikon DS‐Ri1. Cells were imaged sequentially with 4′, 6‐diamidino‐2‐phenylindole (DAPI, ex350/50, em460/50 to detect Hoechst), EGFP ex480/30 nm, em 535/40 nm to detect EGFP‐positive cells, and TRITC (ex540/2 nm, em605/55 nm to detect PI), with a ×10 objective (NIKON Plan Fluor 10X/0.30 WD 16). For each coverslip, five distinct fields of view were acquired. For each field, the ratio of PI‐positive (apoptotic) nuclei over the total number of GFP‐positive nuclei, was calculated. Images were analyzed by using ImageJ (version 1.51k).

### 4.7 Fluorescence confocal microscopy

Cortical neurons were stained as previously described (Rocchi et al., 2019). The list of the used primary antibodies is provided in Table S2. Fluorescently conjugated secondary antibodies were from Molecular Probes (Thermo‐Fischer Scientific). Image acquisition was performed using a confocal microscope (SP8, Leica Microsystems) at ×63 (1.4 NA) magnification. Z‐stacks were acquired every 300 nm, 5 fields/sample. DAPI staining was used to determine the nucleus area.

Analysis of lipofuscin aggregates was conducted by taking advantage of the autofluorescence signal of lipofuscin granules (Jung et al., 2010). Cells were washed once in phosphate‐buffered saline and immediately imaged with 405 nm excitation and long‐pass filtering, excluding wavelengths shorter than 420 nm. The EGFP signal was used to identify transduced cells. The analysis was conducted using ImageJ (version 1.51k). For each set of experiments, all images were acquired using identical exposure settings.

### 4.8 Assay of SA‐β‐gal activity

SA‐β‐gal activity was detected in primary neurons at 14 DIV using the senescence β‐galactosidase staining kit (cell signaling), according to the manufacturer's instructions.

Analysis of reactive oxygen species (ROS), mitochondrial membrane potential, and calcium imaging are described in the supplemental procedures.

### 4.9 Statistical analysis

Results are shown as means ± sem. The number of samples (n) is indicated as follows: n = n’ × n’’, where n’ is the number of replicates (independent experiments performed on the same neuronal preparation) and n’’ is the number of independent neuronal preparations from distinct neonatal REST^GTi^ litters. Normal distribution of data was assessed using the Shapiro–Wilk test. The two‐tailed unpaired Student's *t* test was used to compare two normally distributed sample groups, while either one‐ or two‐way ANOVA followed by the Bonferroni's multiple comparison test was used to compare more than two normally distributed sample groups. For non‐normally distributed datasets, the Mann–Whitney *U* test, the Kruskal–Wallis/Dunn and Dunnett's tests were used. A *p* value <0.05 was considered significant. Statistical analysis was carried out using SigmaStat 13 (Systat software).

## CONFLICT OF INTEREST

The authors declare that they have no conflict of interest.

## AUTHOR CONTRIBUTIONS

AR, EC, and JAK planned and run all the biochemical and molecular biology experiments and analyzed the data; ADF carried out the electron microscopy experiments and analyzed data therefrom; TF generated the REST^GTi^ mouse; AR and FB conceived and funded the study, analyzed the experimental data and wrote the manuscript.

## Supporting information

Supplementary Material

Supplementary Material

Supplementary Material

Supplementary Material

Supplementary Material

Supplementary Material

## Data Availability

Data sharing not applicable to this article as no datasets were generated or analyzed during the current study.
