# Corrigendum to: REST/NRSF deficiency impairs autophagy and leads to cellular senescence in neurons

**DOI:** 10.1111/acel.13900

**Published:** 2023-06-12

Rocchi, A., Carminati, E., De Fusco, A., Kowalska, J.A., Floss, T., Benfenati, F. REST/NRSF deficiency impairs autophagy and leads to cellular senescence in neurons. *Aging Cell*. 2021 Oct;20(10):e13471. 10.1111/acel.13471.

In the published version of the above article, the authors noticed that one of the funding grants from the Italian Ministry of Health was mistakenly omitted. The last sentence in the Acknowledgment section should read as follows:

‘This work was supported by Ministero Istruzione, Università e Ricerca (PRIN‐2017A9MK4R to FB), Compagnia di San Paolo Torino (n. 34760 to FB), and Ministero della Salute Ricerca Finalizzata (GR‐2016‐02363972 and **GR‐2019‐12370176** to AR)’.

The authors apologize for the error.

