# Supplementary material for: REST/NRSF deficiency impairs autophagy and leads to cellular senescence in neurons
Source: Aging Cell. 2021 Sep 14;20(10):e13471. doi: 10.1111/acel.13471 (PMC8520714; doi:10.1111/acel.13471)
Supplement: Supplementary file 6 — Supplementary Material [file ACEL-20-e13471-s006.docx]

*Aging Cell* ACE-20-0574 *R2*

**­­­**

**REST/NRSF DEFICIENCY IMPAIRS AUTOPHAGY AND LEADS TO CELLULAR SENESCENCE IN NEURONS**

**Anna Rocchi^1,2^, Emanuele Carminati^1,3^, Antonio De Fusco^1,2^, Jagoda Aleksandra Kowalska^1^, Thomas Floss ^4^, Fabio Benfenati^1,2^**

^1^ Center for Synaptic Neuroscience and Technology, Istituto Italiano di Tecnologia, Largo Rosanna Benzi 10, 16132 Genova, Italy

^2^ IRCCS, Ospedale Policlinico San Martino, Largo Rosanna Benzi 10, 16132 Genova, Italy

^3^ Department of Experimental Medicine, University of Genova, Viale Benedetto XV, 3, 16132 Genova, Italy

^4^ Helmholtz Zentrum München, Deutsches Forschungszentrum für Gesundheit und Umwelt (GmbH), Ingolstädter Landstr. 1, 85764 Neuherberg, Germany

**SUPPORTING INFORMATION**

**Supplemental Experimental Procedures**

**Analysis of reactive oxygen species (ROS).** ROS levels were measured using the cell-permeable fluorescent indicator CellROX Deep Red (Thermo Fisher, C10422). Superoxide anions, the predominant ROS in mitochondria, were analyzed by MitoSOX Red (Molecular Probes, M36008). Cre-REST and ΔCre-REST neurons were plated in 96-wells black clear-bottom plates (10,000 cells/well). At 14 DIV the CellROX Deep Red or MitoSOX reagent were added to the samples to a final concentration of 5 μM, incubated for 30 min at 37 °C in the dark and then analyzed by Infinite F500 Microplate Reader (Tecan; CellROX Deep RED excitation/emission wavelengths: 640/665 nm; MitoSOX Red excitation/emission 510/585 nm).

**JC-1 dye staining.** The mitochondrial membrane potential (ΔΨm) was detected in primary neurons at 14 DIV using JC-1 dye labeling method (Thermo-Fischer Scientific). JC-1 shifts from green to red with increasing aggregation in mitochondria, thus allowing for a ratiometric, quantitative assessment of the mitochondrial polarization states. Neurons were incubated with 0.25 µg/ml JC-1 at 37 °C, 5% CO_2_, for 30 min in the presence or absence of antimycin A, a complex III inhibitor (40 mM, 1 h), as a positive control. Cells were imaged sequentially for EGFP (ex 480/10 nm, em 505/10 nm), to detect EGFP positive cells, and for the JC1 assay, by recording the green monomer emission (~529 nm) which shifts to red (~590 nm) upon formation of red fluorescent J-aggregates, using a ×63 objective (HC-PL- APO CS2 63X/1.40). For each sample, at least five distinct fields of view were acquired. For each field, the ratio of red aggregates over green monomers of EGFP-positive nuclei, was calculated. Images were analyzed by using ImageJ (version 1.51k).

**Calcium signaling**. Cells were loaded with 1 μg/ml cell-permeable Fura-2 AM (#F1221, Thermo-Fischer Scientific) in the culture medium and maintained for 30 min in the incubator. Cells were then washed with culture medium and incubated for 30 min to allow hydrolysis of the esterified groups. Coverslips were mounted on the imaging chamber and loaded with 0.5 ml of culture medium. Fura-2-loaded cultures were observed with an IX-81 motorized inverted epifluorescence microscope (Olympus, Tokyo, Japan) using a UplanSAPO ×63 1.35 NA oil-immersion objective (Olympus), and recordings were performed from visual fields containing 8 ± 3 infected neurons on average. Samples were excited at 340 and 380 nm by an MT20 Hg–Xe lamp (Olympus). The exciting light was separated from the emitted light using a 395-nm dichroic mirror. Images of fluorescence emission >510 nm were acquired continuously for a maximum of 20 min (200 ms single exposure time) by using a Hamamatsu Orca-ER IEEE1394 CCD camera (Hamamatsu Photonics). The camera operated on 2 × 2 pixel-binning mode, and the imaging system was controlled by an integrating imaging software package (Cell∧R; Olympus). During the analysis, cells were selected by drawing regions of interest around their bodies to reduce the background. Traces were obtained from 340/380 ratios. Peaks with at least 2% of difference with respect to the baseline were considered and their frequency was calculated as the total number of peaks over the recording time.

**Supplementary figures and tables**

**Figure S1.** Assessment of the purity of primary neuronal cultures

**Figure S2.** Cre-REST neurons do not affect the viability of neighboring cells

**Figure S3.** The activation of the senescence program precedes cell death in REST-depleted neurons

**Figure S4.** Transmission electron microscopy in rapamycin-treated neurons

**Figure S5.** Expression of SASP genes in ΔCre-REST neurons upon manipulation of autophagic flux

**Table S1.** List of primers used for real time-PCR experiments.

**Table S2.** List of antibodies used for western blotting and fluorescence confocal microscopy


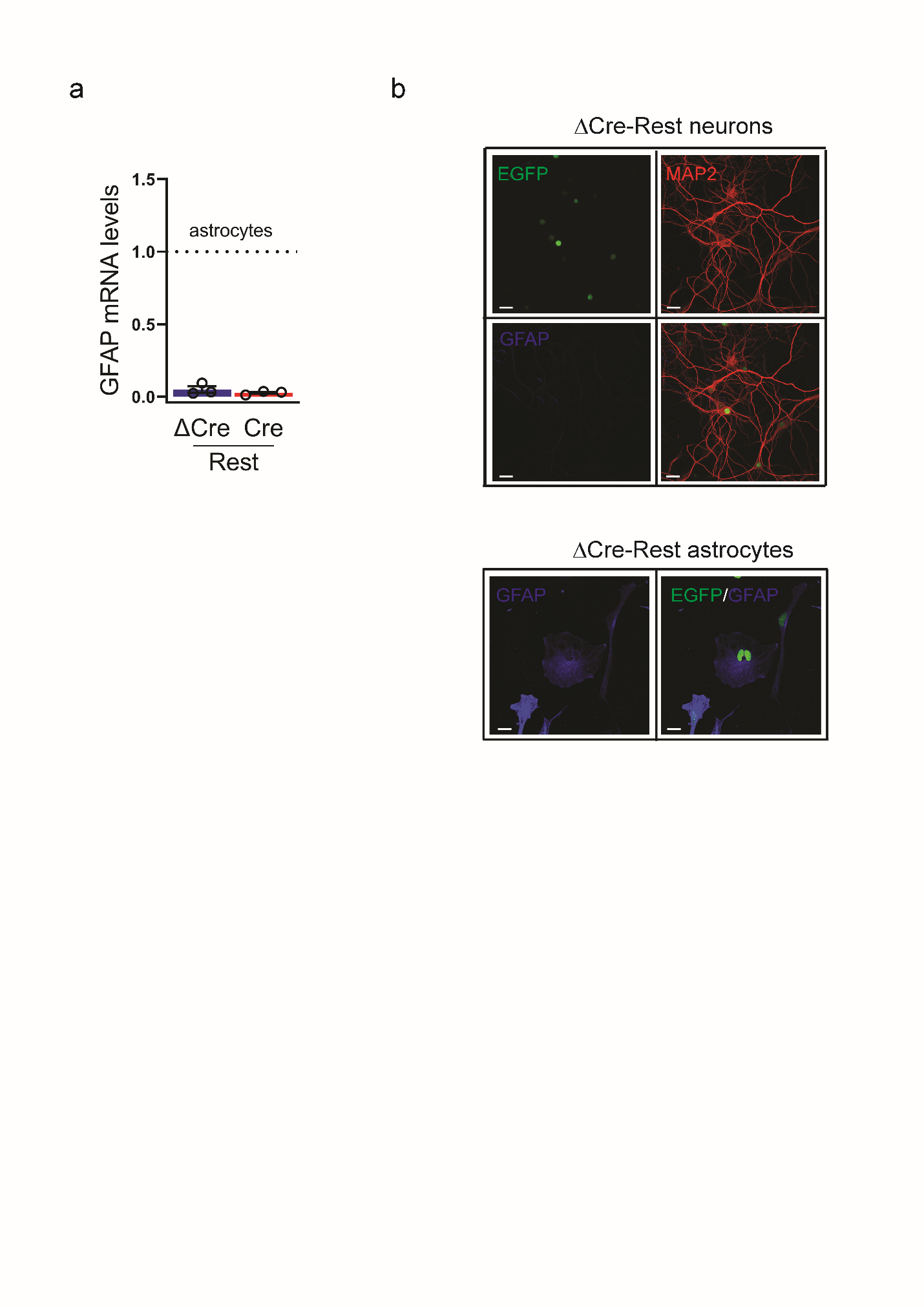


**Figure S1. Assessment of the purity of primary neuronal cultures**

**(a)** qRT-PCR was used to measure mRNA levels of glial fibrillary acidic protein (GFAP) in ΔCre-REST and Cre-REST cortical neurons compared to primary non-transduced astrocytes derived from REST ^GTi/ GTi^ mice. The astrocyte-specific marker GFAP was almost undetectable in neuronal cultures. Gapdh, Actin and GusB were used as control housekeeping genes. Bars show means ± sem from three independent experiments. The dotted line indicates the average GFAP expression level in astrocytes. **(b, upper panels)** Representative images of ΔCre-transduced neurons stained for MAP2 (red) and GFAP (blue). Transduced neurons are EGFP-tagged. n=2 coverslips (5 fields/coverslip) from 2 independent preparations. (b, lower panels) Representative images of ΔCre-transduced astrocytes stained for GFAP (blue), indicating the specificity of GFAP immunostaining. Scale bar, 10 µm


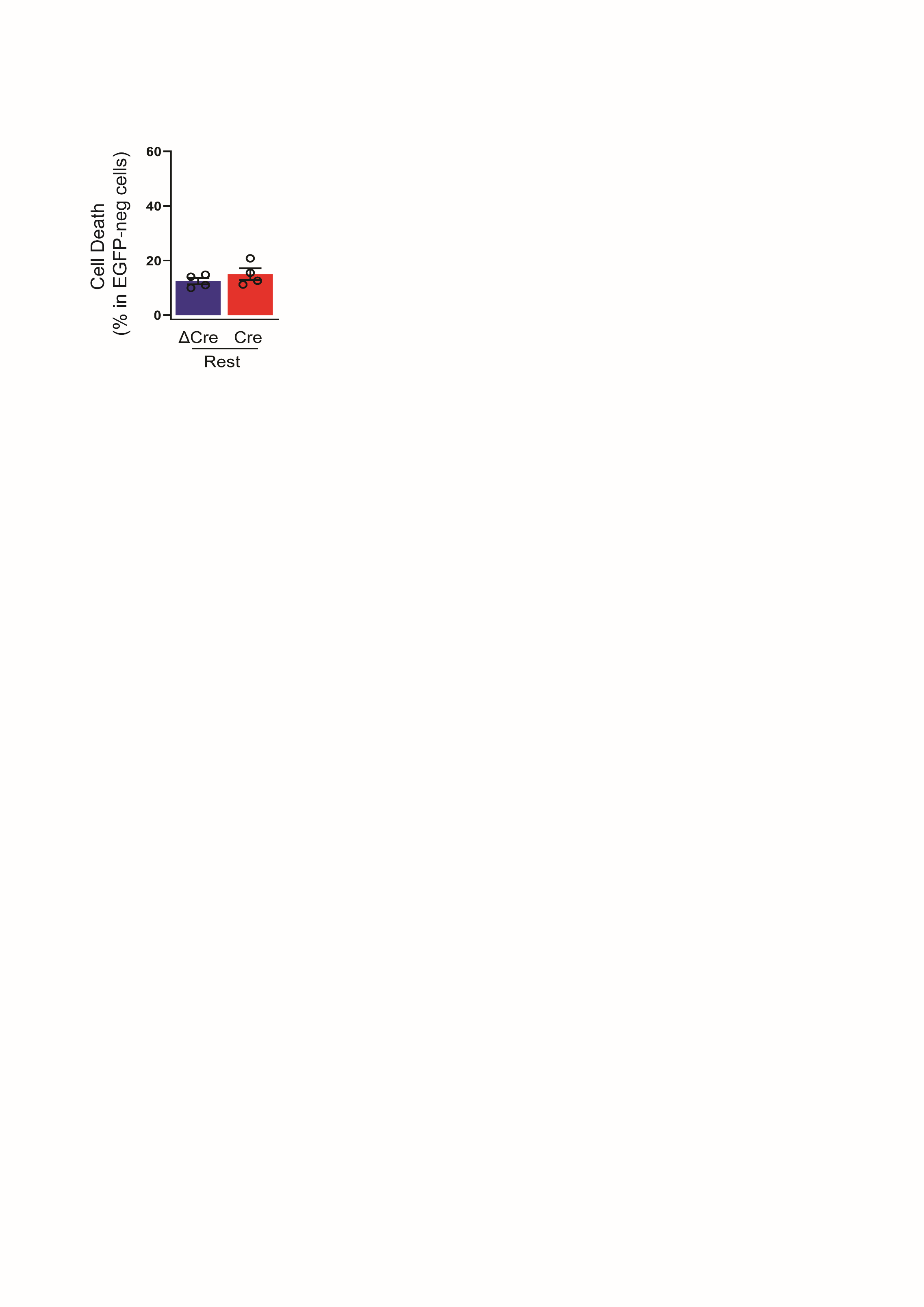


**Figure S2. Cre-REST neurons do not affect the viability of neighboring cells**

Cell death was evaluated by fluorescence microscopy of neurons stained with propidium iodide (PI) for cell death quantification, nuclear EGFP for identification of transduced neurons, and Hoechst 33342 for visualization of all cell nuclei. The bar plot shows the percentage of PI+ cells with respect to the total number of EGFP-negative cells calculated for each experimental group. Graphs show means ± sem from four independent preparations.


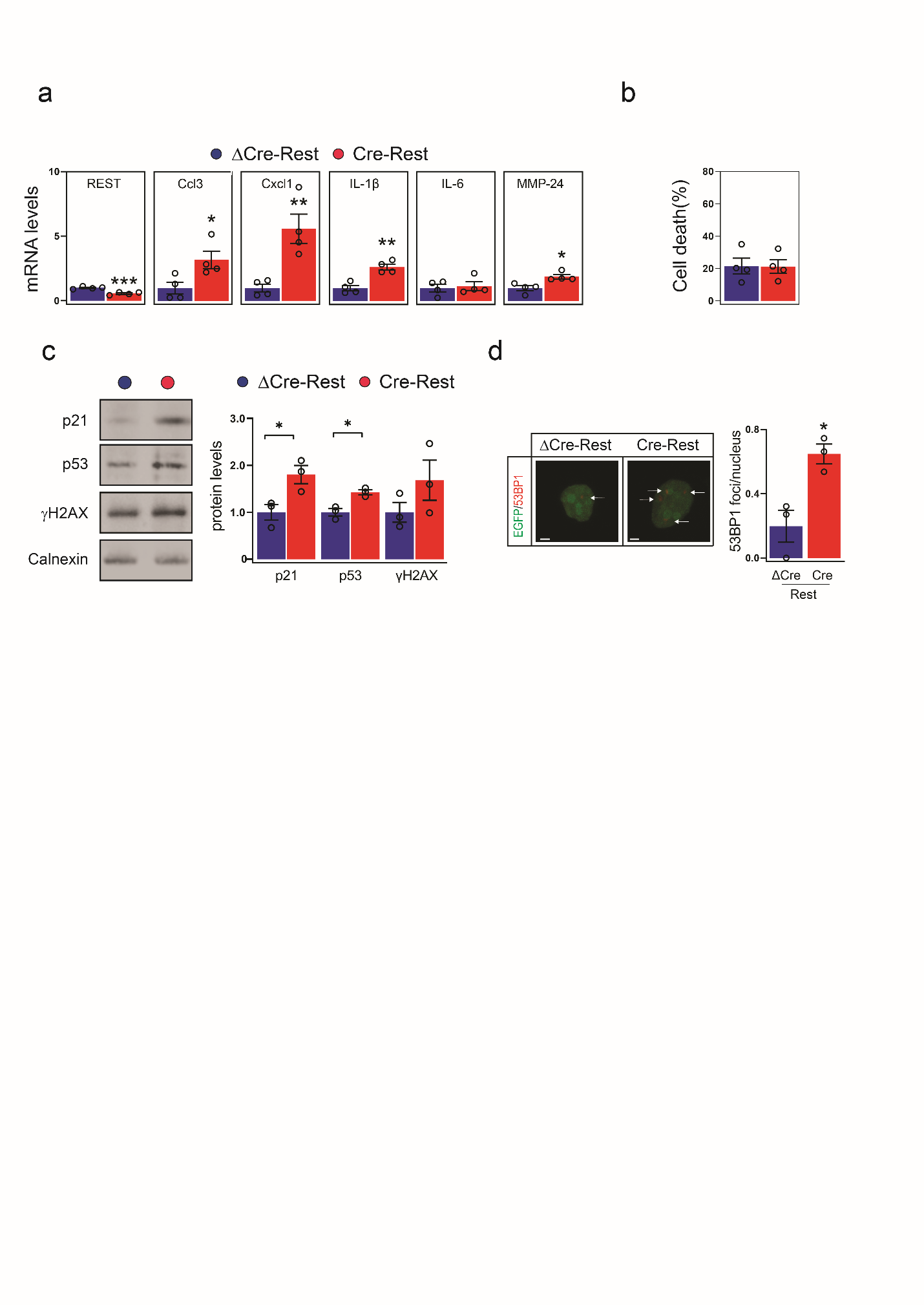


**Figure S3. The activation of the senescence program precedes cell death in REST-depleted neurons**

**(a)** qRT-PCR analysis of mRNA levels of Ccl3, Cxcl1, IL-1β, IL-6, MMP-24 in ΔCre- and Cre-REST cortical neurons at 11 DIV, a stage earlier than that adopted in the experiments shown in Figure 2g (14 DIV). Gapdh, Actin and GusB were used as control housekeeping genes. **(b**) Cell death was evaluated by fluorescence microscopy of neurons stained with propidium iodide (PI) for cell death quantification and nuclear EGFP for identification of transduced neurons. The percentages of PI+ cells with respect to the total number of EGFP+ cells were calculated for each experimental group. (c) Western blotting analysis was used to measure the protein levels of p21, p53 and γH2AX in ΔCre-REST and Cre-REST neurons. Calnexin was used as loading control. (d) Representative images (left) and quantification (right) of 53BP1 foci in ΔCre-REST and Cre-REST neurons. The arrows point to 53BP1 foci. Scale bar, 5 µm. Bars show means ± sem from 3-4 independent preparations. *p <0.05; **p <0.01; ***p <0.001; unpaired two-tailed Student’s *t*-test.


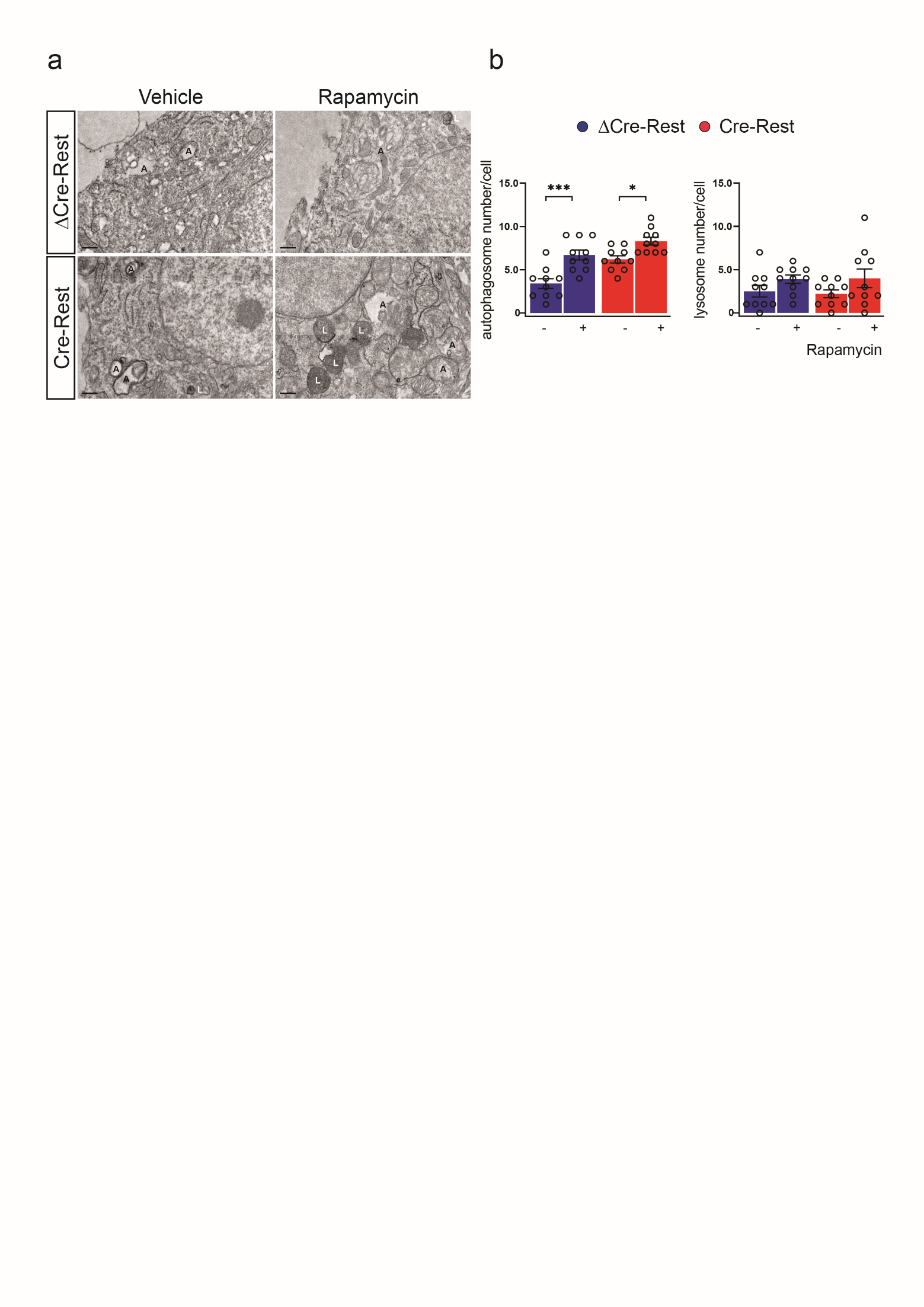


**Figure S4. Transmission electron microscopy in rapamycin-treated neurons**

Representative photomicrographs (**a**) and quantification (**b**) of autophagosomes (labeled with “A”) and lysosomes (marked with an “L”) in ΔCre- and Cre-transduced cultures treated with either vehicle (-) or 30 nM rapamycin (+) for 72 h; Scale bar, 0.5 μm. Graphs show means ± sem. n=10 neurons from 3 independent preparations. *p <0.05; ***p <0.001; two-way ANOVA/Bonferroni’s tests.

**
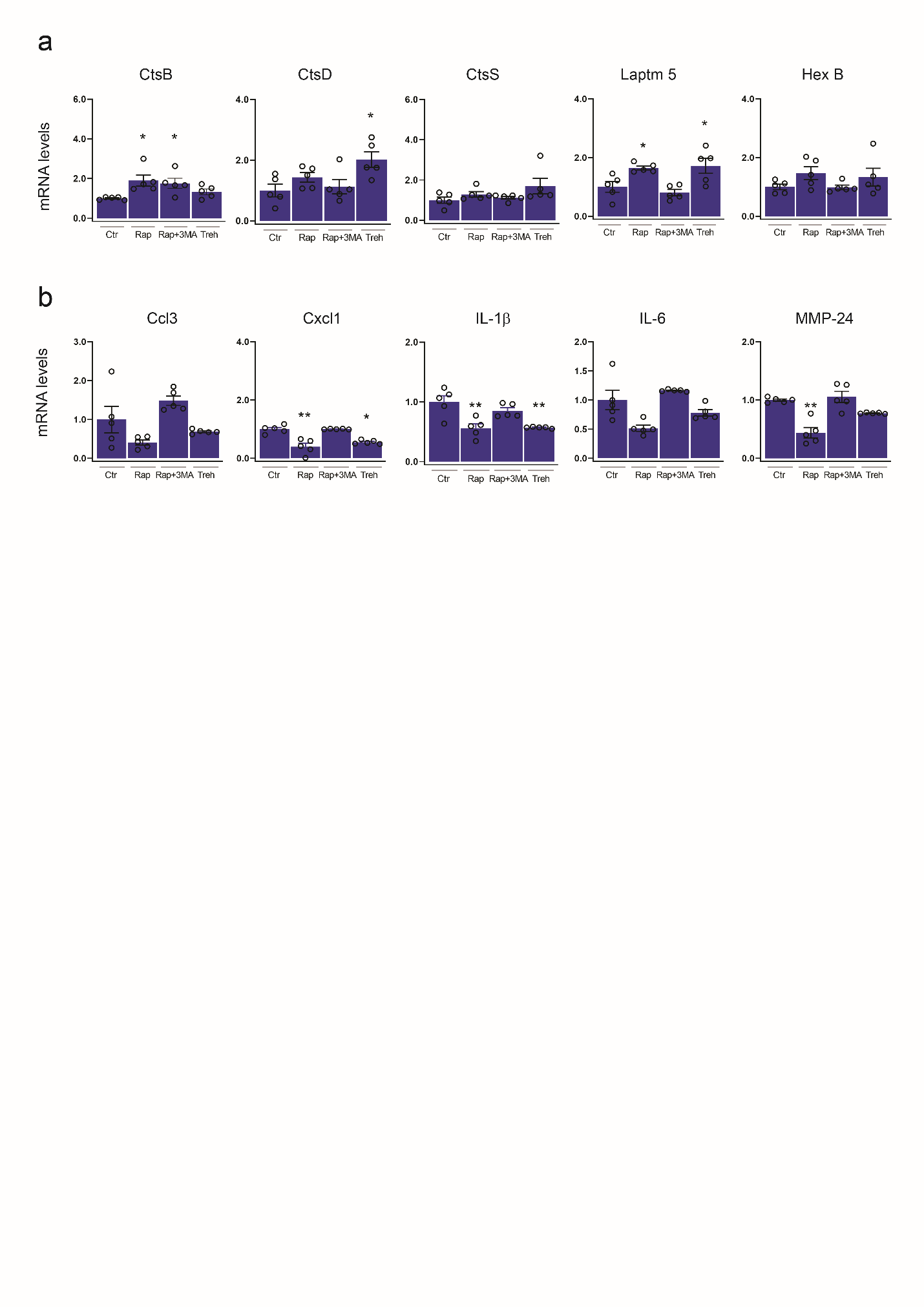
**

**Figure S5. Expression of SASP genes in ΔCre-REST neurons upon manipulation of autophagic flux**

(**a**) The mRNA levels of CtsB, CtsD, CtsS, Laptm 5, Hex B were quantified by qRT-PCR in ΔCre-transduced neurons incubated with vehicle (Ctr), rapamycin (Rap), rapamycin plus 3MA (Rap+3MA) or trehalose for 72 h. (**b**) The mRNA levels of Ccl3, Cxcl1, IL-1β, IL-6, MMP-24 were quantified by qRT-PCR in Cre-transduced neurons incubated with vehicle (Ctr), rapamycin (Rap), rapamycin plus 3MA (Rap+3MA) or trehalose for 72 h. Gapdh, Actin and GusB were used as control housekeeping genes. Graphs show means ± sem with superimposed individual points from five independent preparations (blue, ΔCre-REST; red, Cre-REST). *p <0.05; **p <0.01; ***p <0.001; one-way ANOVA/Dunnett’s tests versus vehicle

| **Table S1. List of primers used for real time-PCR experiments.** | | |
| --- | --- | --- |
| **Gene Symbol** | **Forward sequence** | **Reverse sequence** |
| Gapdh | TAGACAAAATGGTGAAGGT | AGTTGAGGTCAATGAAGG |
| Actin | AAGTGGTTACAGGAAGTCC | ATAATTTACACAGAAGCAATGC |
| GusB | TCGGGCTGGTGACCTACTGGATTTCTG | GTTGGCACTGGGAACCTGAAGTTGACC |
| REST | GAACCACCTCCCAGTATG | CTTCTGACAATCCTCCATAG |
| Cdkn1a | CGAGAACGGTGGAACTTTGAC | CAGGGCTCAGGTAGACCTTG |
| Ccna2 | TGCAAACTGTAAGGTTGAAAGC | TGTAGAGAGCCAAGTGGAAGG |
| Ccnb1 | TGCATTTTGCTCCTTCTCAA | CAGGAAGCAGGGAGTCTTCA |
| Map1lc3a | CACTGCTCTGTCTTGTGTAGGTTG | TCGTTGTGCCTTTATTAGTGCATC |
| Sqstm1 | CCCAGTGTCTTGGCATTCTT | AGGGAAAGCAGAGGAAGCTC |
| CtsB | CTGCTGAAGACCTGCTTA | AATTGTAGACTCCACCTGAA |
| CtsD | TCTGTTCTGTGGTTCCTC | GACAAGACAATCCATCTCAG |
| CtsS | GATGGGTGCTCTGAGAAT | GCAATGTCCGATTAGAGTATG |
| Laptm5 | GCCATTTACCACATAGTCAT | GCATCTTGAAGAACCTACAG |
| Hex B | GCTCCTGGTCTCCATTAC | CGGCTACTGGTTCTTGTA |
| Tp53 | GCTACCTGAAGACCAAGAA | TCATAAGACAGCAAGGAGAG |
| Cdkn2a | GAACTCTTTCGGTCGTACCC | CGAATCTGCACCGTAGTTGA |
| Ccl3 | CTTGCTGTTCTTCTCTGT | AATAGTCAACGATGAATTGG |
| Cxcl1 | CCACTGTGCTAGTAGAAG | GACGACTTTCAATAAATAACTG |
| IL-1β | AAACGGTTTGTCTTCAAC | GTGAAGTCAATTATGTCCTG |
| IL-6 | ATCAGTTTGTGGACATTCC | TTGTCAGTTCTTCGTAGAGA |
| MMP-24 | CTGCACTGCTCTTAGAAA | TTAGGAACTGTCAGATGTG |
| Gfap | GCTCCAAGATGAAACCAA | GATTCAACCTTTCTCTCCAA |

| **Table S2.** List of antibodies used for western blotting and fluorescence confocal microscopy | | | |
| --- | --- | --- | --- |
| **Antibodies** | **Source** | **Identifier** | **Application** |
| rabbit anti-REST | Millipore | 07-579 | WB |
| rabbit anti-calnexin | Enzo Life Sciences | ADI-SPA 860 | WB |
| rabbit anti-p21 | Cell Signaling | 2947 | WB |
| mouse anti-ubiquitin | Santa Cruz | 8017 | WB |
| rabbit anti-phospho-Ser240/244-S6 | Cell Signaling | 2215 | WB |
| mouse anti-total S6 | Cell Signaling | 2317 | WB |
| rabbit anti-p62 | Sigma-Aldrich | P0067 | WB/IF |
| mouse anti-LC3 | Nanotools | 231 | WB |
| rabbit anti-lamin B1 | NovusBio | NBP1-19804 | WB |
| rabbit anti-Foxo1 | Cell Signaling | 2880 | WB |
| mouse anti-cyclinA2 | Sigma-Aldrich | C4710 | WB |
| rabbit anti-p16 | Cell Signaling | 80772 | WB |
| rabbit anti-phospho-Ser 139 -Histone H2A.X | Cell Signaling | 2577 | WB |
| rabbit anti-Atg8 | Rockland | 200-401-H57 | IF |
| mouse anti-Lap2 | BD Transduction Laboratories | 611000 | IF |
| rabbit anti-53BP1 | Bethyl Laboratories, Inc | A300-272A-T | IF |
| rabbit anti-Lamp1 | Sigma-Aldrich | L1418 | IF |
| rabbit anti-Map2 | Abcam | Ab32454 | IF |
| mouse anti-GFAP | Sigma-Aldrich | G 3893 | IF |
